# Does the taste matter? Taste and medicinal perceptions associated with five selected herbal drugs among three ethnic groups in West Yorkshire, Northern England

**DOI:** 10.1186/1746-4269-3-21

**Published:** 2007-05-03

**Authors:** Andrea Pieroni, Bren Torry

**Affiliations:** 1 SCH Group, Department of Social Sciences, Wageningen University, Postbus 8060, NL-6700 DA Wageningen, The Netherlands; 2 Division of Pharmacy Practice, School of Life Science, University of Bradford, Richmond Bd., Richmond Rd., Bradford, BD7 1DP, West Yorkshire, UK

## Abstract

In recent years, diverse scholars have addressed the issue of the chemosensory perceptions associated with traditional medicines, nevertheless there is still a distinct lack of studies grounded in the social sciences and conducted from a cross-cultural, comparative perspective. In this urban ethnobotanical field study, 254 informants belonging to the Gujarati, Kashmiri and English ethnic groups and living in Western Yorkshire in Northern England were interviewed about the relationship between taste and medicinal perceptions of five herbal drugs, which were selected during a preliminary study. The herbal drugs included cinnamon (the dried bark of *Cinnamomum verum*, Lauraceae), mint (the leaves of *Mentha *spp., Lamiaceae), garlic (the bulbs of *Allium sativum*, Alliaceae), ginger (the rhizome of *Zingiber officinale*, Zingiberaceae), and cloves (the dried flower buds of *Syzygium aromaticum*, Myrtaceae).

The main cross-cultural differences in taste perceptions regarded the perception the perception of the spicy taste of ginger, garlic, and cinnamon, of the bitter taste of ginger, the sweet taste of mint, and of the sour taste of garlic.

The part of the study of how the five selected herbal drugs are perceived medicinally showed that TK (Traditional Knowledge) is widespread among Kashmiris, but not so prevalent among the Gujarati and especially the English samples. Among Kashmiris, ginger was frequently considered to be helpful for healing infections and muscular-skeletal and digestive disorders, mint was chosen for healing digestive and respiratory troubles, garlic for blood system disorders, and cinnamon was perceived to be efficacious for infectious diseases.

Among the Gujarati and Kashmiri groups there was evidence of a strong link between the bitter and spicy tastes of ginger, garlic, cloves, and cinnamon and their perceived medicinal properties, whereas there was a far less obvious link between the sweet taste of mint and cinnamon and their perceived medicinal properties, although the link did exist among some members of the Gujarati group.

Data presented in this study show how that links between taste perceptions and medicinal uses of herbal drugs may be understood as bio-cultural phenomena rooted in human physiology, but also constructed through individual experiences and culture, and that these links can therefore be quite different across diverse cultures.

## Background

The interest of a few medical ethnobotanists and ethnopharmacologists appears to have shifted in the recent past from the documentation and bio-evaluation of traditionally used herbal drugs and the search for plant-based "miracle cures" to investigations aimed at a better understanding of the use and medicinal perceptions of botanicals within human societies.

In this regard, Etkin and Elisabetzy [1] recently issued the following sharply worded warning:

The most demanding work for the future will be to build theoretical capacity in ethnopharmacology. To date, only a handful of researchers have contributed to this development, exploring such issues as:

*a. How local environmental knowledge both undergrids and emerges from co-evolutionary people-plant-landscape relations*.

*b. How the apprehension and management of resources is culturally constructed and socially transacted in ways that influence knowledge asymmetries and health disparities*.

*c. How the multicontextual use of plants (in medicine, food, cosmetics, etc.) impacts health, as well as the conservation of cultural and biological diversity*.

This change in focus for ethnopharmacological research has two main trajectories: how humans' organoleptic perceptions may have been, and may continue to be, crucial criteria for their selection of medicine or *medicinal foods *[2-5]; and how the use of botanical medicines has changed and evolved, especially after population displacements and migrations [6-9].

Over the past two decades, diverse scholars have addressed the issue of the chemosensory perceptions associated with traditional medicines. Johns and Keen in their pioneering work on the taste perceptions and classifications of Aymara in Bolivia [10] have shown how the unique taste taxonomy of the Aymara (an expanded nomenclature for bitter substances and the absence of a clear concept of sourness) and the association of pleasantness with sweetness could have an adaptive significance. In 1998, Brett and Heinrich hypothesized on the importance of the cultural meaning of organoleptic properties determined by plant phytochemicals [11]. In 2000, Casagrande clearly showed in his studies on taste and cognition among the Tzeltal Maya that the use of medicinal plants cannot be predicted based on taste alone. He suggests that the role of taste is more likely to be mnemonic rather than chemical-ecological, hence the combination of plant attributes with illness experiences could explain the occurrence of prototypical groups of plants used to treat specific groups of illnesses [12].

Similar conclusions were reached by a recent food ethnobotanical study conducted among Albanians in Southern Italy, where findings showed that the influence of the bitter taste perception in the food versus medicinal classification of wild botanicals, and the existence of prototypical ethnolinguistic categories of weedy food plants, seem to be the result of morphological, functional and also chemosensoric perceptions of bitterness [2].

Leonti et al. [3] and Gollin [13] have confirmed the chemosensory hypothesis by showing a strong link between perceived organoleptic properties (smell and taste) of specific plants and their use as medicines among the Popoluca in Mexico and the Kenyah Leppo' Keh of Borneo, respectively. Leonti et al. have also suggested that the doctrine of signatures could have represented a crucial mnemonic aid to help users remember the application assigned to a given plant.

Shepard introduced in his study of two Amazonian societies the new concept of "sensory ecology" to define a new theoretical perspective, in which sensations can be understood as bio-cultural phenomena rooted in human physiology, and also constructed through individual experiences and culture [14]. These findings are important because Shepard reinforces how organoleptic properties can change over time and across and between different cultures.

In order to contribute to a greater understanding of how the perceptions and multi-contextual use of commonly used herbal drugs are culturally constructed, this paper presents the results of an urban medical ethnobiological study conducted in Western Yorkshire among three ethnic groups. Given the occurrence of a remarkable proportion of South-Asian population, Western Yorkshire and Bradford in particular represent in fact very appropriate multi-cultural arenas for investigating cross-cultural health beliefs.

The study aimed to explore whether *taste retains an importance in determining *emic *medicinal perceptions of botanicals.*

More specifically, the objectives of the research were:

• to explore the taste and medicinal perception of botanicals commonly used as medicines by three ethnic groups living in West Yorkshire;

• to evaluate the occurrence of eventual links between taste perception and the specific use of these drugs;

• to determine whether relevant differences existed among the three groups;

• to propose explanations for these differences (if they exist).

## Methods

### Overall approach

The study used both qualitative and quantitative methods of data collection and analysis. It was conducted in two stages; a preliminary study and a main study. Ethical approval for the study was granted by the University of Bradford Ethics Committee.

It was intended that a questionnaire would be used to collect data from three different ethnic groups living in West Yorkshire regarding their taste perceptions of five selected herbal drugs, which would to be known to and used by all three groups.

In order to choose the most appropriate herbal drugs and ethnic groups for this study, and also to pilot the questionnaire, a preliminary study was conducted.

### Preliminary study

The preliminary data collection involved a survey across five different ethnic groups of people living in the Leeds and Bradford area. Between eight and twelve informants were selected from each of the following ethnic groups: English, Caribbean, Chinese, Gujarati, and Kashmiri. The informants were asked to free-list ten herbal drugs they commonly used and to describe their tastes. These selections were then categorised and cross referenced; the aim was to identify 5 herbs commonly used by at least three ethnic groups.

During this stage a structured questionnaire was designed and piloted for use in the main study. The aim of the questionnaire was to collect demographic variables in order to enhance comparative analysis.

The results showed major differences in the use of herbal drugs between the Chinese and Caribbean groups and the other groups. Since the aim of the project was to assess cross-cultural differences in taste perceptions and the medicinal use of commonly used herbal drugs, *whose use had to be shared across all the ethnic communities*, only the three "closest" groups were considered for the main study: Kashmiris (K), Gujaratis (G), and English (E).

From 46 freelisted herbal drugs, only those five, which were more commonly cited across all three groups were considered. They included: cinnamon (the dried bark of *Cinnamomum verum*, Lauraceae), mint (the leaves of *Mentha *spp., Lamiaceae), garlic (the bulbs of *Allium sativum*, Alliaceae), ginger (the rhizome of *Zingiber officinale*, Zingiberaceae), and cloves (the dried flower buds of *Syzygium aromaticum*, Myrtaceae).

A similar type of analysis was carried out for the terms used to describe the taste perceptions associated with the named herbal drugs. From all cited terms (40),, the expressions selected included those which were more ubiquitously used to define the taste of the five selected botanicals; these were "bitter", "sweet", "salty", "sour", "spicy", and "bland". The term "hot" was not considered, because in many cases it was used as a synonym for "spicy".

### The three selected communities

The Kashmiri population in Northern England migrated to the UK from the 1950s onwards, from the highly disputed region of Kashmir. Kashmiri is a Dardic language belonging to the Indo-Iranian subgroup of the Indo-European languages. It is estimated that there are approximately five million Kashmiri speakers worldwide [15]. The Kashmiri ethnic group is the largest minority ethnic group living in the Bradford area.

The Gujarati people in Northern England were for the most part born in India or Eastern Africa (to families, who had previously migrated from Western India), and came to Britain in the late 1960s and 1970s. Gujarati is an Indo-Iranian language, which is part of the greater Indo-European language family. It is estimated there are 46 million speakers of Gujarati worldwide [15].

Finally, the English autochthonous population in Western Yorkshire belong to the North English group, speaking varieties of non-rhotic Northern English (Yorkshire dialects).

### Main study

A sample of 274 members from the English, Gujarati and Kashmiri communities were randomly selected in Bradford, Leeds and Dewsbury, and interviewed in public locations, i.e. shopping centres, mosques or local community centres over a period of three weeks in March 2006, using a questionnaire that was piloted during the preliminary study. Interviewees were specifically asked to define the taste of each of the five herbal drugs selected for this survey, and to name their perceived medicinal properties or uses. The age, gender and ethnic background of each participant was recorded.

**Figure 1:**
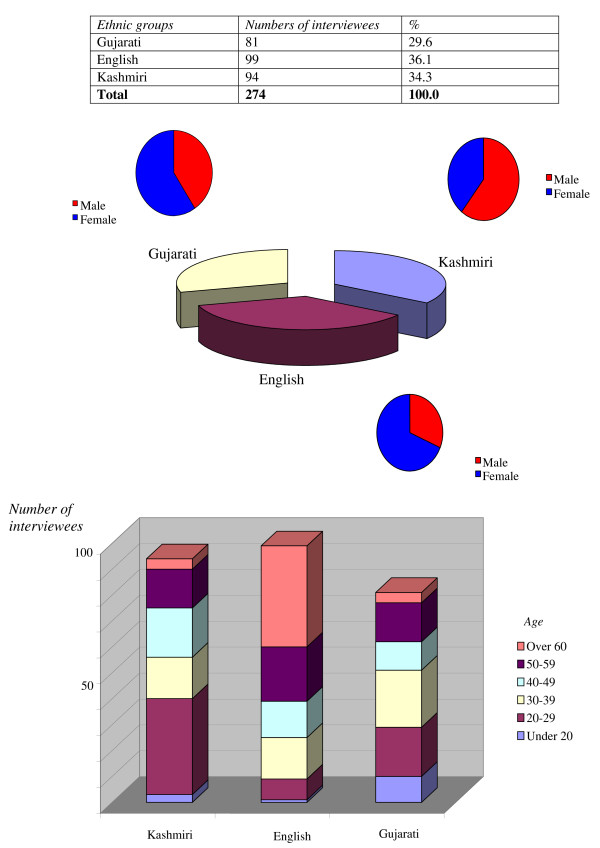
Characteristics of the chosen sample of informants.

The characteristics of the chosen sample are reported in Figure 1, which graphically illustrates a reasonable balance in the number of people recruited to the study across the three different groups; 36% English (n = 99), 34% Kashmiri participants (n = 94) and 30% Gujarati participants (n = 81). Overall 118 participants were male (43%) and 156 participants female (57%). Women were a majority representation within two ethnic groups (English and Kashmiri) whereas males were a majority in the Gujarati ethnic group. Participant age was recorded across 6 different categories.

At the close of each interview participants were invited to mention anything else they had in mind regarding their taste perceptions or their knowledge of herbal drug use that could bring to light new pieces of information.

### Data analysis

Data was analysed using descriptive statistics and cross tables in the 'Statistics Package for Social Sciences' (SPSS). Graphs were produced in Microsoft Excel (MS Excel).

## Results and discussion

### Taste perceptions associated with the selected herbal drugs

**Figure 2:**
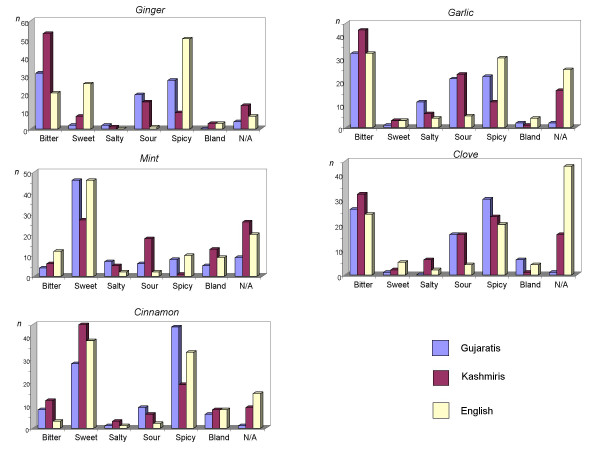
Taste perceptions associated with the five selected herbal drugs across the three ethnic groups.

Figure 2 shows the taste perceptions associated with each of the five selected herbal drugs among the three ethnic groups. If considering only those quotations given by more than 20% of all participants, from the graphs it is evident that the main cross-cultural differences in taste perceptions were as follows:

• the perception of the *spicy taste of ginger, garlic, and cinnamon *(in all three cases, there was only a relatively small sample of the Kashmiri and a large sample of English informants describing these herbal drugs as spicy);

• the perception of the *bitter taste of ginger *(a relatively large majority of Kashmiri participants perceived this herbal drug as bitter);

• the perception of the *sweet taste of mint *(relatively small proportion of Kashmiri informants);

• the perception of the *sour taste of garlic *(relatively small sample of the English participants).

### Medicinal perceptions associated with the selected herbal drugs

**Figure 3:**
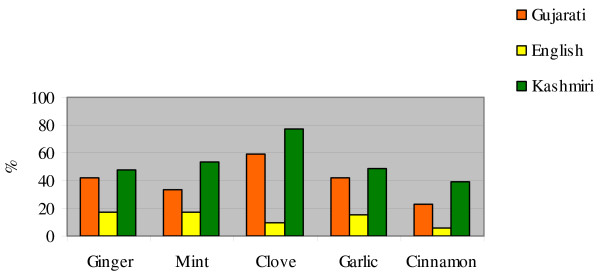
Comparison of the overall medicinal perception of the five selected herbal drugs across the three ethnic groups.

Figure 3 and Table 1 give details of medicinal perceptions associated with the selected herbal drugs across the three ethnic groups. We can clearly see that:

**Table 1:** Specific medicinal perceptions/uses of the five herbal drugs across the three ethnic groups

*Uses*	*English*					*Kashmiri*					*Gujarati*				
	Ginger	Mint	Garlic	Clove	Cinnamon	Ginger	Mint	Garlic	Clove	Cinnamon	Ginger	Mint	Garlic	Clove	Cinnamon
Blood System Disorders	0	0	8	0	0	1	0	39	1	0	3	0	26	0	2
Digestive System Disorders	8	12	1	0	4	16	37	3	3	2	3	16	0	0	8
Endocrine System Disorders	3	0	0	0	2	2	0	0	0	1	1	0	0	0	1
Infections	3	1	5	1	0	12	3	3	2	11	18	7	6	2	4
Muscular-Skeletal System Disorders	2	0	1	0	0	14	0	1	1	0	1	0	0	0	0
Respiratory System Disorders	0	4	0	0	0	0	10	0	0	0	2	3	0	0	0
Pain Relieving	0	0	0	9	0	1	0	0	65	3	2	0	1	46	3
Mental Disorders	0	0	0	0	0	0	0	0	0	0	4	1	0	0	0
Others	0	1	0	0	0	1	3	0	0	2	12	20	15	8	24

• ginger is perceived by many more Kashmiris than by members of either of the other two groups as being helpful for healing infections and muscular-skeletal and digestive disorders;

• mint is perceived by more Kashmiris than by members of either of the other two groups as being helpful for healing digestive and respiratory troubles;

• garlic is perceived by more Kashmiris than by members of either of the other two groups as being helpful for healing blood system disorders;

• cinnamon is perceived by more Kashmiris than by members of either of the other two groups as being helpful against infectious diseases.

• overall, the Kashmiris had the highest number of participants, proportionately, perceiving medicinal uses for each of the five herbs; the English participants had the lowest perception of medicinal use in all cases.

The fact that more Kashmiris have strong perceptions about the medicinal properties of herbal drugs could perhaps be explained by the great resilience of their Traditional Medicine (TM).

### The link between specific taste and medicinal perceptions

**Figure 4:**
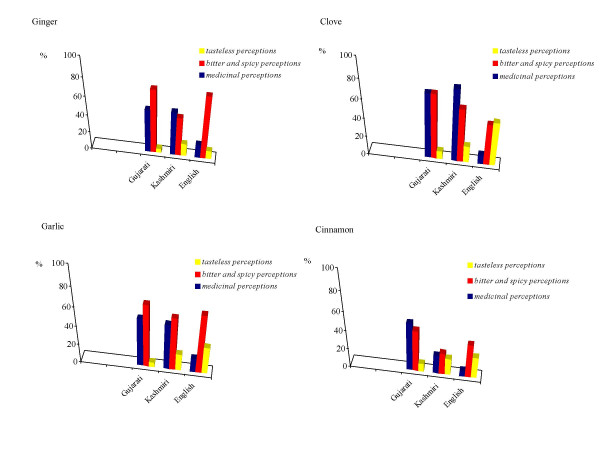
Relations between bitter and spicy taste and medicinal perceptions for ginger, garlic, clove, and cinnamon.

Figure 4 shows how for the Gujarati and Kashmiri groups a strong link exists between the frequency of the perception of bitter and spicy tastes of ginger, garlic, clove, and cinnamon, and the frequency of perceived medicinal perceptions. Values related to "no taste perceptions" in Figure 3 are the sum of answers reporting a "bland" perception or no answers at all – N/A (even it could be that informants did not know how to describe a particular flavour, we felt that often the N/A response was associate in the mind of the interviewees to the lack of a specific taste perception).

**Figure 5:**
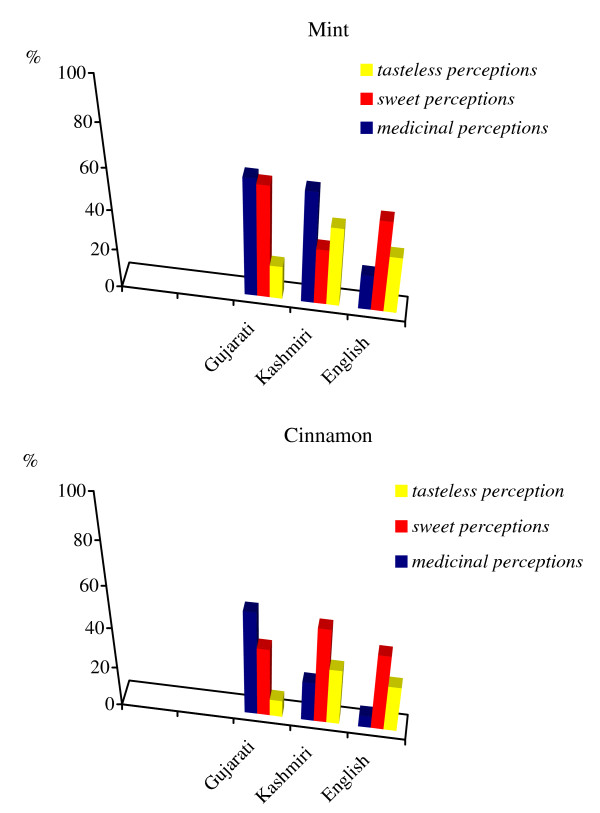
Relations between sweet and medicinal perceptions for mint and cinnamon.

Figure 5 shows how there is a far less evident link between the sweet taste and the medicinal perceptions of mint and cinnamon among the Gujarati group, whereas the Kashmiri group gave a much more complex response.

The lack of a clear link between taste and medicinal perceptions among the autochthonous English group can possibly be explained by the low degree of knowledge about the medicinal properties of herbal drugs that the informants of this group displayed.

### Age, gender, and taste perceptions

**Figure 6:**
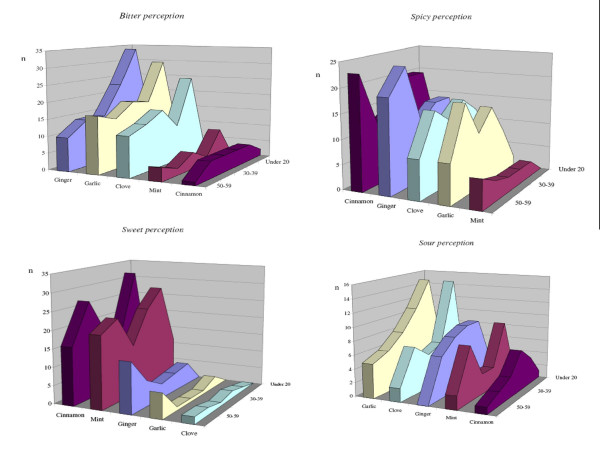
Relations between bitter, spicy, sweet and sour herbal drug's taste perceptions and age of the interviewees.

Figure 6 shows how bitter, spicy, sour, sweet perceptions relate to age. While younger interviewees tended more frequently to define the taste of the selected drugs as "bitter" and "sour", the elderly more frequently reported them as being "spicy". Interviewees in the middle generations less frequently cited perceptions of sweetness. Unfortunately, due to the inconsistency in representation of all categories of age in the English ethnic group further analysis was considered unreliable.

**Figure 7:**
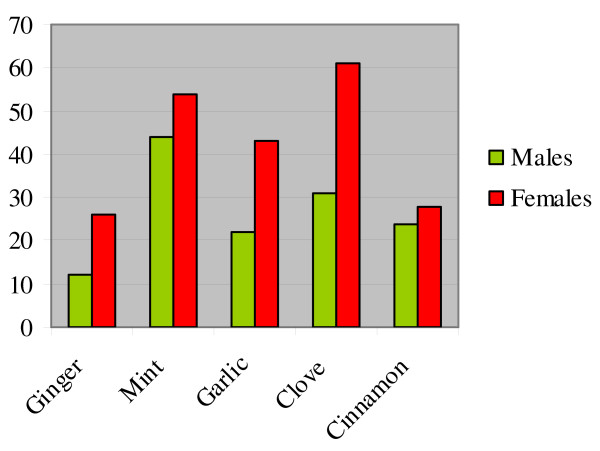
Percentage of males and females perceiving the herbal drugs as "tasteless".

Analysis of gender differences in taste showed that the perception of the "tastelessness" (including the two categories of bland and not applicable) of ginger, mint, garlic, clove and cinnamon was consistently perceived at a proportionately higher rate in females than in males (Figure 7).

## Conclusion

Data reported in this study clearly show that links between taste perceptions and the medicinal uses of herbal drugs may be quite different across diverse cultures.

We feel that these phenomena can be much better explained using Shepard's "sensory ecology" approach, in which sensations can be understood as bio-cultural phenomena rooted in human physiology, *but also constructed through individual experiences and culture *[14]. Hence assumptions regarding the existence of presumed universal roles played by chemosensory perception in the medicinal perceptions of botanicals [13] should be very critically verified by further studies, since chemosensory is probably only one of the key criteria that people – especially in urban and acculturated societies – use to "categorise" widely available herbal drugs.

A major role in these phenomena is surely played also by individual and collective experiences and the cultural history of botanicals too may also explain for example in our study why South-Asians (Kashmiris and Gujaratis) seemed to more frequently link for ginger, garlic, clove, and cinnamon bitter and spicy tastes with medicinal perceptions.

However, our findings could have been influenced or limited by our specific research settings:

• our study was based on perceptions recorded for "only" five herbal drugs that are widely known by all three communities. This small number was necessary in order for us to be able to manage the research with an acceptable sample of interviewees and in a reasonable time span; nevertheless by targeting so few drugs, we have clearly limited the possibility of including less known herbal drugs. In other words, our data refer to remedies, whose "cultural history" of use has been extraordinarily dense and have instead ignored botanicals, which are much less used, or whose cultural history of use has been less determinant;

• while, on the one hand, the inclusion of the English group has permitted an interesting comparison between migrant and autochthonous communities, in contrast it has highlighted the problem of the relevant erosion of TK (Traditional Knowledge) about medicines and herbal drugs within this group. This in turn means that we could have "artificially" overestimated for the English group the disjunction between specific tastes and medicinal perceptions;

• the different age composition of the three cultural groups (especially in the case of the English sample, which was overrepresented by elderly people), could have had an effect on the results of interviewees' taste perceptions regarding the herbal drugs, although this influence could have been compensated by the effect that may have occurred as a consequence of the major erosion of TK among the same group.

It could be worthwhile to observe future trajectories in research on the role played by taste in the medicinal perceptions of herbal drugs, and to conduct further studies like this one but with broader samples of informants and including more botanicals.

**Table 2:** Taste perceptions of herbal drugs in relation to the gender of the informants (in bold are reported the only data showing significant differences in the perceptions among the genders of the interviewees).

*Herb*	*Taste*	*Men*		*Women*	
		
		*n*	*%*	*n*	*%*
Ginger	Bitter	48	17.5	56	20.4
	Sweet	13	4.7	21	7.7
	Salty	3	1.1	0	0
	Spicy	42	15.3	44	16.1
	Sour	16	5.8	19	6.9
	Bland	2	0.7	4	1.5
	N/A	6	2.2	18	6.6

Mint	Bitter	7	2.6	15	5.5
	Sweet	56	20.5	63	23.1
	Salty	7	2.6	7	2.6
	Spicy	10	3.7	9	3.3
	Sour	11	4	15	5.5
	Bland	14	5.1	13	4.8
	N/A	19	7	36	13.2

Garlic	Bitter	46	16.8	60	21.9
	Sweet	4	1.5	3	1.1
	Salty	12	4.4	9	3.3
	Spicy	31	11.3	32	11.7
	Sour	21	7.7	28	10.2
	Bland	2	0.7	5	1.8
	N/A	12	44	31	11.3

Clove	**Bitter**	**35**	**12.8**	**47**	**17.2**
	Sweet	3	1.1	5	1.8
	Salty	5	1.8	23	1.1
	**Spicy**	**28**	**16.4**	**45**	**40.5**
	**Sour**	**23**	**8.4**	**13**	**4.7**
	Bland	6	2.2	5	1.8
	**N/A**	**15**	**5.5**	**45**	**16.4**

Cinnamon	Bitter	11	4	12	4.4
	Sweet	44	16.1	67	24.5
	Salty	2	0.7	3	1.1
	Spicy	42	15.3	54	19.7
	Sour	10	3.6	7	2.6
	Bland	14	5.1	8	2.9
	N/A	7	2.6	18	6.6
